# Aromatase inhibitors for short stature in male children and adolescents treated with growth hormone: a meta-analysis of randomized controlled trials

**DOI:** 10.1186/s12887-024-05301-0

**Published:** 2024-12-18

**Authors:** Kan Wang, Fei Ye, Da-Yan Wang, Pan-Jian Lai, Lin-Qian Zhang

**Affiliations:** 1 Jinhua Maternity and Child Health Care Hospital, Jinhua Women and Children’s Hospital, Jinhua, 322199 China; 2 First Department of Neurology, Affiliated Jinhua Hospital, Jinhua Municipal Central Hospital, Zhejiang University School of Medicine, Jinhua, 321000 China

**Keywords:** Aromatase inhibitor, Growth hormone, Short stature, Meta-analysis

## Abstract

Whether the addition of aromatase inhibitors (AIs) to recombinant human growth hormone (rhGH) could yield additional benefit for short stature is controversial. We aimed to assess the effects of combined AIs and rhGH versus those of rhGH alone for short stature using a meta-analytic approach. The PubMed, Embase, and the Cochrane library electronic databases were searched systematically for eligible randomized controlled trials (RCTs) from inception until December 2021. The pooled effect estimates (weighted mean difference [WMD] and odds ratio [OR]) with 95% confidence intervals (CIs) were calculated using a random-effects model. Eight RCTs that provided data on 433 participants were selected. The addition of AIs to rhGH, compared with rhGH alone, resulted in higher growth velocity (WMD: 3.19 cm/year; 95% CI: 2.75–3.63; *P* < 0.001), higher predicted adult height (WMD: 5.50 cm; 95% CI: 3.52–7.49; *P* < 0.001), and younger bone age (WMD: -0.80 years; 95% CI: -1.06–-0.54; *P* < 0.001). There were no significant differences between the groups for insulin-like growth factor I (WMD: 0.85 nmol/L; 95% CI: -2.08–3.79; *P* = 0.569), serum estradiol level (WMD: -19.19 pmol/L; 95% CI: -46.25–7.88; *P* = 0.165), and serum testosterone level (WMD: 14.88 nmol/L; 95% CI: -14.13–43.88; *P* = 0.315). There was no significant difference between the groups for the risk of adverse events (OR: 1.08; 95% CI: 0.44–2.66; *P* = 0.873). This study found that the addition of AIs to rhGH had greater benefits for growth velocity, predicted adult height, and bone age. The safety profiles were comparable.

**Supplementary Information:**

The online version contains supplementary material available at 10.1186/s12887-024-05301-0.

## Introduction

Short stature in childhood is a development disorder that occurs frequently and is the most common cause for referral to pediatric endocrinologists [1]. A variety factors may cause short stature, including growth hormone deficiency, intrauterine growth retardation, genetic disorders, hypothyroidism, celiac disease, and nutritional deficiency. Although infants born small for gestational age is an important characteristic of short stature, the majority of these infants may experience catch-up growth in the first year [2, 3]. Children who have a short stature are often treated differently, which can cause different degrees of psychological problems [4], and effective treatment for children and adolescent should be implemented.

Nowadays, recombinant human growth hormone (rhGH) is the most commonly used agent for short stature as it may stimulate the production of an insulin-like growth factor-1 (IGF-1) in the liver and other organs and act on the chondrocytes in the epiphyseal plate to promote the synthesis and metabolism of protein, which play an important role in promoting bone growth [5]. However, the responsiveness to rhGH therapy may be affected by genetics, biochemical variability, and diagnoses [6, 7]. Two common aromatase inhibitors (AIs) of anastrozole and letrozole may block conversion of testosterone to estradiol and thereby slow advancement of bone age. The inhibitory potencies of anastrozole and letrozole have been shown to be nearly 97% and 99%, respectively [8]. Whether the addition of AIs to rhGH could yield additional benefit for short stature remains controversial. We therefore performed a meta-analysis of randomized controlled trials (RCTs) to assess the effects of combined AIs and rhGH versus that of rhGH alone for short stature.

## Methods

### Data sources, search strategy, and selection criteria

The Preferred Reporting Items for Systematic Reviews and Meta-Analysis guidelines was used as a guide in the reporting of this study (S1 Checklist) [9]. RCTs that compared the effects of combined AIs and rhGH versus that of rhGH alone for short stature in male children and adolescents were eligible for inclusion in our study. No restrictions were placed on the published language and status. The PubMed, Embase, and the Cochrane library electronic databases were searched systematically for eligible trials throughout December 2021, using the following text word or Medical Subject Heading terms: “Aromatase Inhibitors” AND “Body Height OR Growth Disorders” AND “Randomized controlled trial.” Moreover, the website of http://www.ClinicalTrials.gov was searched for trials that were already completed, but not yet published. We also searched the reference lists of relevant reviews, manually, to identify any new eligible trial.

Studies were included if they met the following criteria: (1) Participants: individuals younger than 18.0 years who presented with short stature; (2) Interventions: AIs and rhGH; (3) Control: rhGH; and (4) Outcomes: growth velocity, predicted adult height, bone age, IGF-I, serum estradiol, serum testosterone, and adverse events; and (5) Study design: the study had to have RCT design. The literature search and study selection were performed independently by two reviewers, and conflicts between reviewers were settled with mutual discussions until a consensus was reached.

### Data collection and quality assessment

Two reviewers abstracted the following information independently: first author’s surname, publication year, country, sample size, mean age, bone age, height, puberty stage, testosterone, intervention, control, treatment duration, and reported outcomes. The quality of the included trials were assessed using the Jadad scale, which is based on randomization, concealment of treatment allocation, blinding, completeness of follow-up, and use of intention-to-treat analysis. The scale system for each trial ranged from 0 to 5 [10]. The data extraction and quality assessment were conducted independently by two reviewers, and any inconsistencies between the reviewers were settled by group discussions.

### Statistical analysis

A weighted mean difference (WMD) and an odds ratio (OR) with 95% confidence interval (CI) was assigned as an effect estimate for the continuous and categories variables, respectively. The pooled analyses used a random-effects model to consider the underlying variations across the included trials [11, 12]. Heterogeneity across the included trials for each investigated outcome was assessed using *I*^*2*^ and the Cochran Q statistic, with significant heterogeneity being defined as *I*^*2*^ > 50.0% or *P* < 0.10 [13, 14]. Sensitivity analyses were performed through the sequential removal of individual trials to assess the robustness of the pooled conclusion [15]. Thereafter, subgroup analyses were performed for growth velocity, predicted adult height, and bone age on the basis of publication year, country, intervention, and study quality, and an interactive *P* test was used to compare the differences between the subgroups [16]. Publication bias was assessed using both qualitative and quantitative approaches, including the funnel plot, Egger, and Begg tests [17, 18]. The *P* value for all the pooled results were two-sided, and the inspection level was 0.05. Software STATA (version 10.0; Stata Corporation, College Station, TX) was used to perform all the statistical analyses in this study.

## Results

### Literature search and study selection

**Fig. 1:**
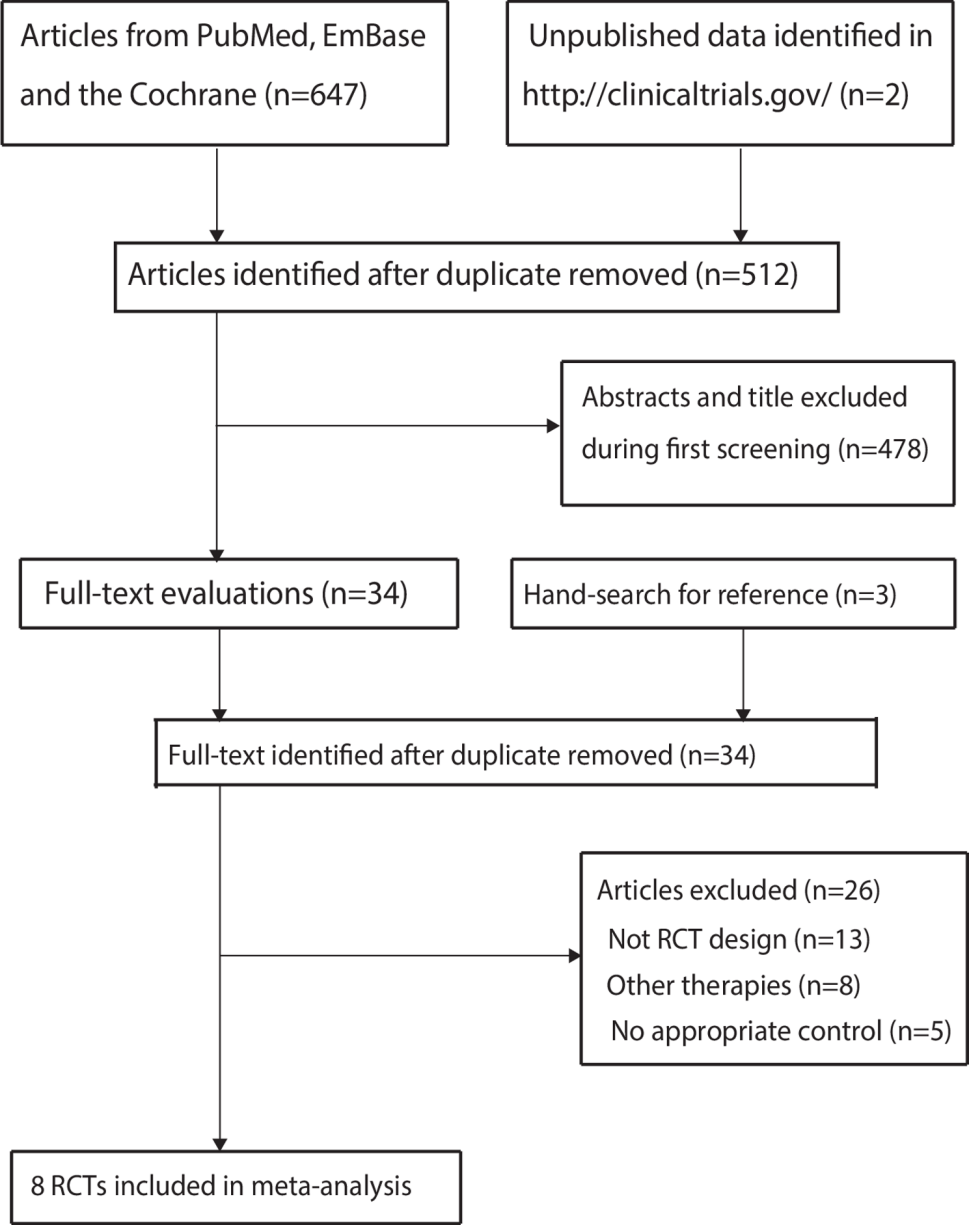
PRISMA flowchart for the literature search and study selection

A total of 647 articles were identified from the electronic searches, and 512 studies were retained after the duplicate articles were removed. Thereafter, 478 studies were removed because these studies had irrelevant titles, and the remaining 34 studies were retrieved for full-text evaluations. After detailed evaluations, 26 studies were excluded due to: not having an RCT design (*n* = 13), not using combined therapies (*n* = 8), and no appropriate control present (*n* = 5). A review of the website of http://www.ClinicalTrials.gov and the reference lists of relevant reviews did not reveal any new eligible trials. Finally, a total of eight RCTs were selected for the final meta-analysis (Fig. 1) [19–26]. 

### Characteristics of included studies

The baseline characteristics of the included studies and their participants are summarized in Table 1. In the eight included trials, 433 individuals were participants, and the sample sizes ranged from 17 to 151. Two trials were performed in Finland, two in the USA, and the remaining four trials in China. Five trials used letrozole and rhGH as interventions, two used anastrozole or letrozole and rhGH as interventions, and the remaining one trial used anastrozole and rhGH as an intervention. The quality of the included trials were assessed using the Jadad scale; three trials had a score of four scores and were considered to be of high quality. Three trials had a score of 3, and two trials had a score of two, which was regarded as low quality.

**Table 1:** The baseline characteristics of identified studies and involved participants

Study	Country	Sample size (treated/control)	Age (years)	Bone age (years)	Height (cm)	Puberty stage (G/*P*)	Testosterone (nmol/L)	Intervention	Control	Treatment duration	Study quality
Wickman 2001 [19]	Finland	22 (10/12)	15.1 (15.2/15.0)	12.8 (13.1/12.6)	153.4 (155.3/151.9)	2/1; 2/1	10.8 (9.5/11.9)	Letrozole + rhGH	rhGH	18.0 months	4
Hero 2006 [20]	Finland	17 (9/8)	15.0 (15.2/14.8)	12.8 (13.0/12.5)	NA	2/1; 2/1	9.9 (8.3/11.7)	Letrozole + rhGH	rhGH	12.0 months	3
Mauras 2008 [21]	USA	52 (26/26)	14.0 (13.8/14.2)	13.6 (13.7/13.4)	150.7 (149.7/151.6)	NA	NA	Anastrozole + rhGH	rhGH	36.0 months	4
Mauras 2016 [22]	USA	51 (26/25)	14.0 (14.0/14.1)	12.8 (12.7/12.9)	144.4 (144.5/144.2)	NA	NA	Anastrozole/letrozole + rhGH	rhGH	36.0 months	4
Wang 2019 [23]	China	36 (18/18)	12.6 (12.6/12.6)	14.0 (14.1/13.8)	125.0 (124.6/125.3)	NA	NA	Letrozole + rhGH	rhGH	24.0 months	3
Kong 2020 [24]	China	151 (108/43)	12.5 (12.5/12.3)	13.9 (13.9/14.0)	NA	NA	12.7 (12.4/13.3)	Anastrozole/letrozole + rhGH	rhGH	20.2 months	2
Song 2020 [25]	China	48 (24/24)	11.3 (11.1/11.4)	11.9 (11.8/12.0)	119.0 (119.5/118.4)	NA	NA	Letrozole + rhGH	rhGH	24.0 months	3
Wang 2020 [26]	China	56 (28/28)	12.3 (12.0/12.5)	14.0 (14.1/13.8)	116.0 (115.8/116.2)	NA	NA	Letrozole + rhGH	rhGH	24.0 months	2

*GH: Growth hormone

### Growth velocity

**Fig. 2:**
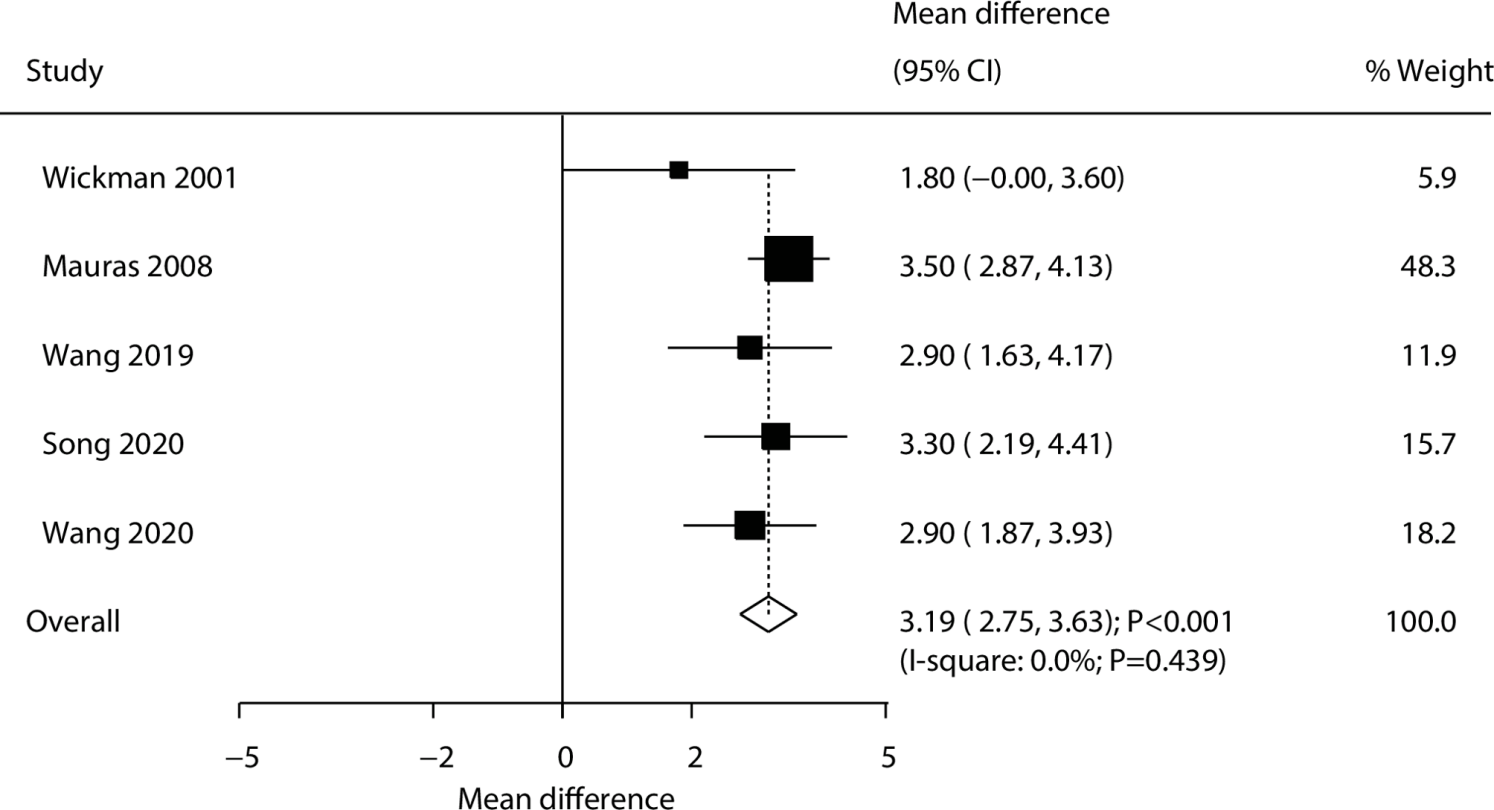
Effect of the combination of AIs and rhGH versus rhGH alone on the change of growth velocity (cm/year)

A total of 5 trials reported the effects of combined AIs and rhGH versus rhGH alone on the change of growth velocity. During the treatment period, we noted that the combination of AIs and rhGH was associated with elevated growth velocity compared with rhGH alone (WMD: 3.19 cm/year; 95% CI: 2.75–3.63; *P* < 0.001; Fig. 2), and no evidence of heterogeneity was observed (*I*^*2*^ = 0.0%; *P* = 0.439). Sensitivity analyses indicated that the pooled conclusion was robust and not altered by excluding any particular trial (S1 Fig). The results of the subgroup analyses were consistent with those of the overall analysis of all the groups (Table 2). Although the Begg test indicated no significant publication bias (*P* = 0.221), the Egger test found a potential significant publication bias for growth velocity (*P* = 0.031) (S2 Fig).

**Table 2:** Subgroup analyses for investigated outcomes

Outcomes	Factors	Subgroup	Number of studies	WMD and 95%CI	*P* value	Heterogeneity (%)	Q statistic	Interaction *P* test
Growth velocity	Publication year	Before 2010	2	2.87 (1.26 to 4.48)	< 0.001	67.2	0.081	0.536
		2010 or after	3	3.04 (2.39 to 3.68)	< 0.001	0.0	0.848	
	Country	Europe or USA	2	2.87 (1.26 to 4.48)	< 0.001	67.2	0.081	0.536
		China	3	3.04 (2.39 to 3.68)	< 0.001	0.0	0.848	
	Intervention	Anastrozole	1	3.50 (2.87 to 4.13)	< 0.001	-	-	0.176
		Letrozole	4	2.90 (2.29 to 3.50)	< 0.001	0.0	0.586	
	Study quality	High	2	2.87 (1.26 to 4.48)	< 0.001	67.2	0.081	0.536
		Low	3	3.04 (2.39 to 3.68)	< 0.001	0.0	0.848	
Predicted adult height	Publication year	Before 2010	3	5.68 (5.01 to 6.36)	< 0.001	0.0	0.677	< 0.001
		2010 or after	5	5.70 (2.91 to 8.49)	< 0.001	88.4	< 0.001	
	Country	Europe or USA	4	4.26 (1.41 to 7.12)	0.003	92.9	< 0.001	0.374
		China	4	7.37 (2.47 to 12.26)	0.003	83.8	< 0.001	
	Intervention	Anastrozole	1	5.70 (5.02 to 6.38)	< 0.001	-	-	< 0.001
		Letrozole	5	7.88 (4.56 to 11.19)	< 0.001	42.5	0.138	
		Anastrozole or letrozole	2	3.02 (1.16 to 4.88)	0.001	86.0	0.007	
	Study quality	High	3	4.43 (1.27 to 7.59)	0.006	95.3	< 0.001	0.418
		Low	5	6.63 (2.65 to 10.62)	0.001	78.7	0.001	
Bone age	Publication year	Before 2010	1	-1.30 (-2.13 to -0.47)	0.002	-	-	0.213
		2010 or after	5	-0.74 (-1.02 to -0.47)	< 0.001	0.0	0.507	
	Country	Europe or USA	2	-0.86 (-1.52 to -0.20)	0.011	53.0	0.144	0.755
		China	4	-0.84 (-1.18 to -0.49)	< 0.001	0.0	0.452	
	Intervention	Anastrozole	1	-1.30 (-2.13 to -0.47)	0.002	-	-	0.151
		Letrozole	3	-1.23 (-1.92 to -0.54)	< 0.001	0.0	0.617	
		Anastrozole or letrozole	2	-0.65 (-0.95 to -0.36)	< 0.001	0.0	0.743	
	Study quality	High	2	-0.86 (-1.52 to -0.20)	0.011	53.0	0.144	0.755
		Low	4	-0.84 (-1.18 to -0.49)	< 0.001	0.0	0.452	

### Predicted adult height

**Fig. 3:**
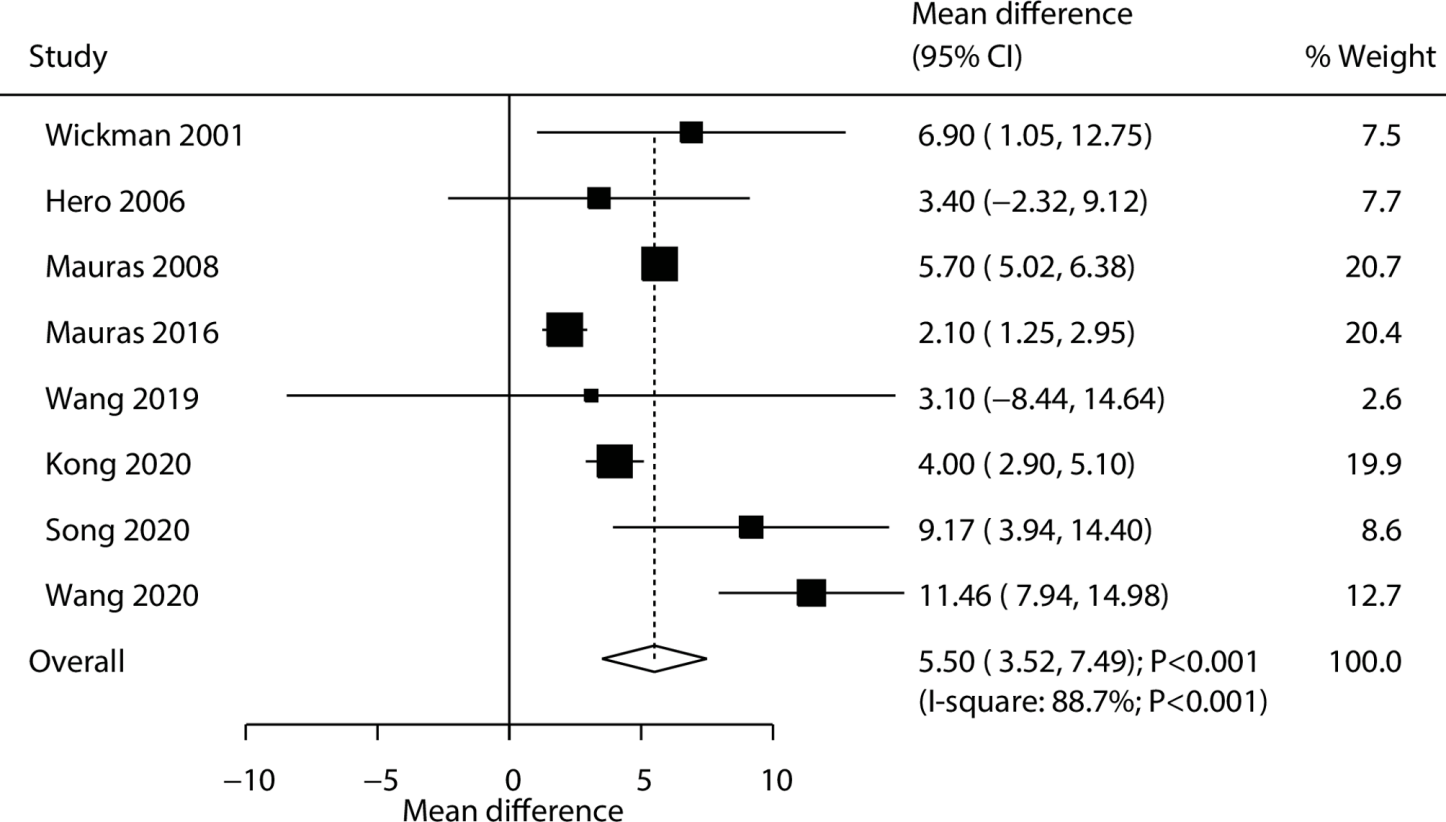
Effect of the combination of AIs and rhGH versus rhGH alone on the change of predicted adult height (cm)

All trials reported the effects of the combination of AIs and rhGH versus rhGH alone on the changes in the predicted adult height. The addition of AIs to rhGH increased the predicted adult height significantly compared with rhGH alone (WMD: 5.50 cm; 95% CI: 3.52–7.49; *P* < 0.001; Fig. 3), and significant heterogeneity was observed among the included trials (*I*^*2*^ = 88.7%; *P* < 0.001). The pooled conclusion of stability was not changed after the sequential removal of a single trial (S3 Fig). The subgroup analyses found significant differences between the addition of AIs to rhGH and rhGH alone in all groups, while the publication year (*P* < 0.001) and intervention (*P* < 0.001) may have affected the treatment effect between the groups (Table 2). There was no significant publication bias for the predicted adult height (*P* value for Egger: 0.565; *P* value for Begg: 0.902; S4 Fig).

### Bone age

**Fig. 4:**
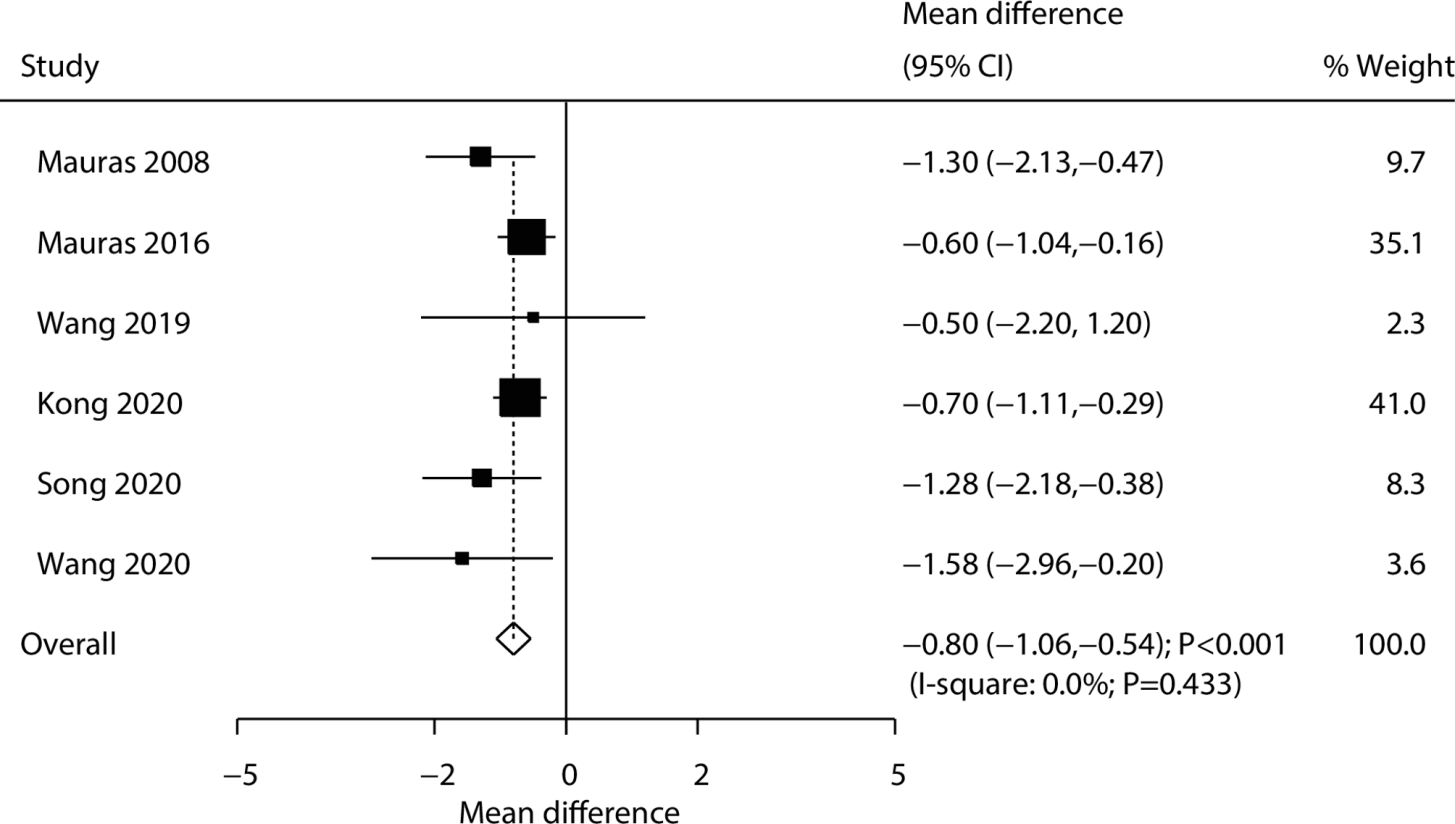
Effect of the combination of AIs and rhGH versus rhGH alone on the change of bone age (years)

A total of six trials reported on the effect of the combination of AIs and rhGH versus rhGH alone on the changes in bone age. We noted that the combination of AIs and rhGH was associated with younger bone age compared with rhGH alone (WMD: -0.80 years; 95% CI: -1.06–-0.54; *P* < 0.001; Fig. 4), and there was no evidence of heterogeneity across the included trials (*I*^*2*^ = 0.0%; *P* = 0.433). The sensitivity analyses found that the pooled conclusion was robust and not altered by the sequential removal of an individual trial (S5 Fig). The results of the subgroup analyses were consistent with those of the overall analysis of all the groups, and the differences between the subgroups were not statistically significant (Table 2). No significant publication bias for bone age was observed (*P* value for Egger: 0.155; *P* value for Begg: 0.707; S6 Fig).

### Other outcomes

**Fig. 5:**
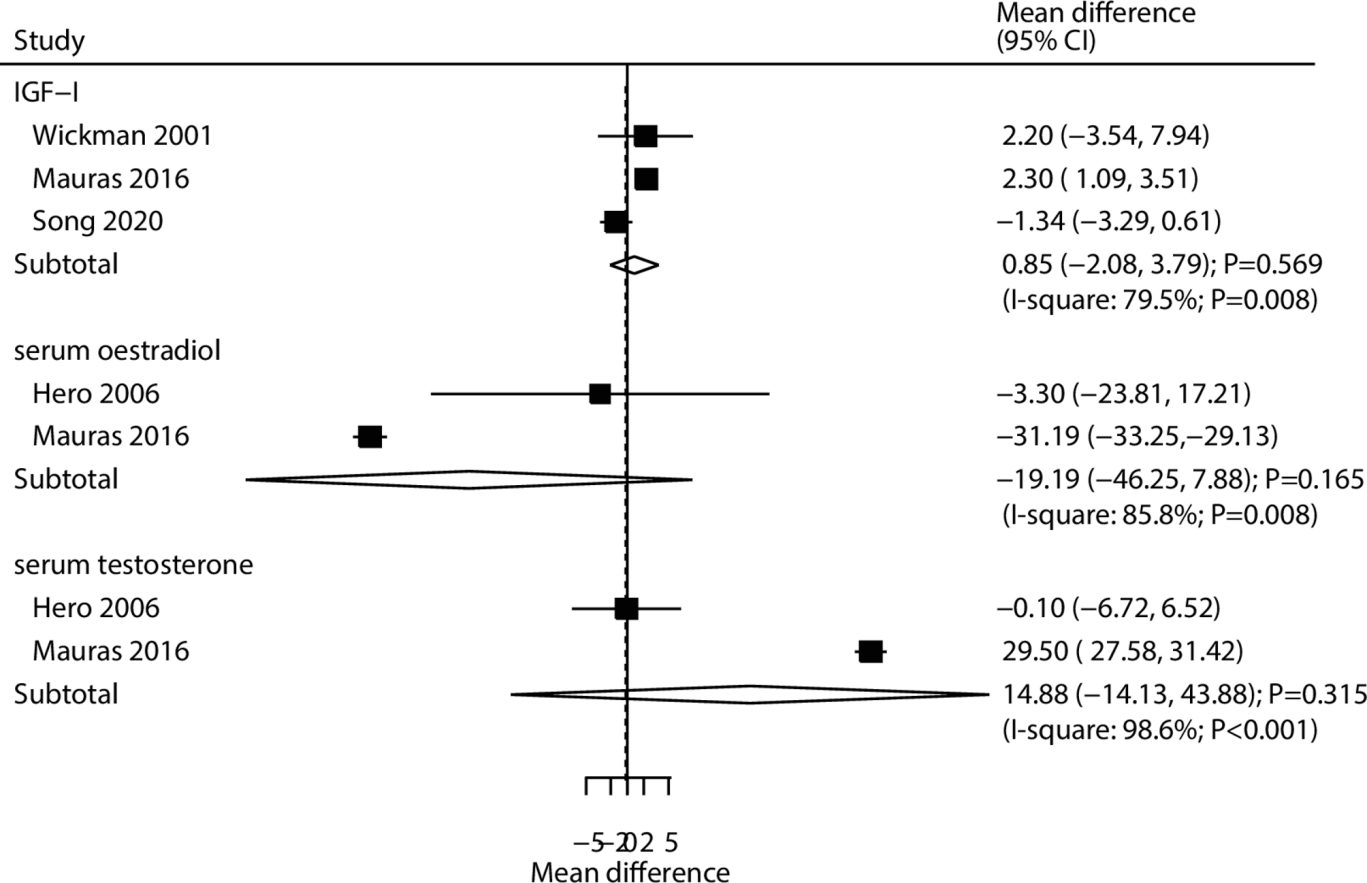
Effect of the combination of AIs and rhGH versus rhGH alone on the changes of IGF-I (nmol/L), serum estradiol (pmol/L), and serum testosterone (nmol/L)

The breakdown of the effects of combined AIs and rhGH versus rhGH alone on the changes of IGF-I, serum estradiol, and serum testosterone levels was reported in three, two, and two trials, respectively (Fig. 5). There were no significant differences between the addition of AIs to rhGH and rhGH alone in the changes of IGF-I (WMD: 0.85 nmol/L; 95% CI: -2.08–3.79; *P* = 0.569), serum estradiol (WMD: -19.19 pmol/L; 95% CI: -46.25–7.88; *P* = 0.165), and serum testosterone (WMD: 14.88 nmol/L; 95% CI: -14.13–43.88; *P* = 0.315), and significant heterogeneity was observed for IGF-I (*I*^*2*^ = 79.5%; *P* = 0.008), serum estradiol (*I*^*2*^ = 85.8%; *P* = 0.008), and serum testosterone (*I*^*2*^ = 98.6%; *P* < 0.001).

**Fig. 6:**
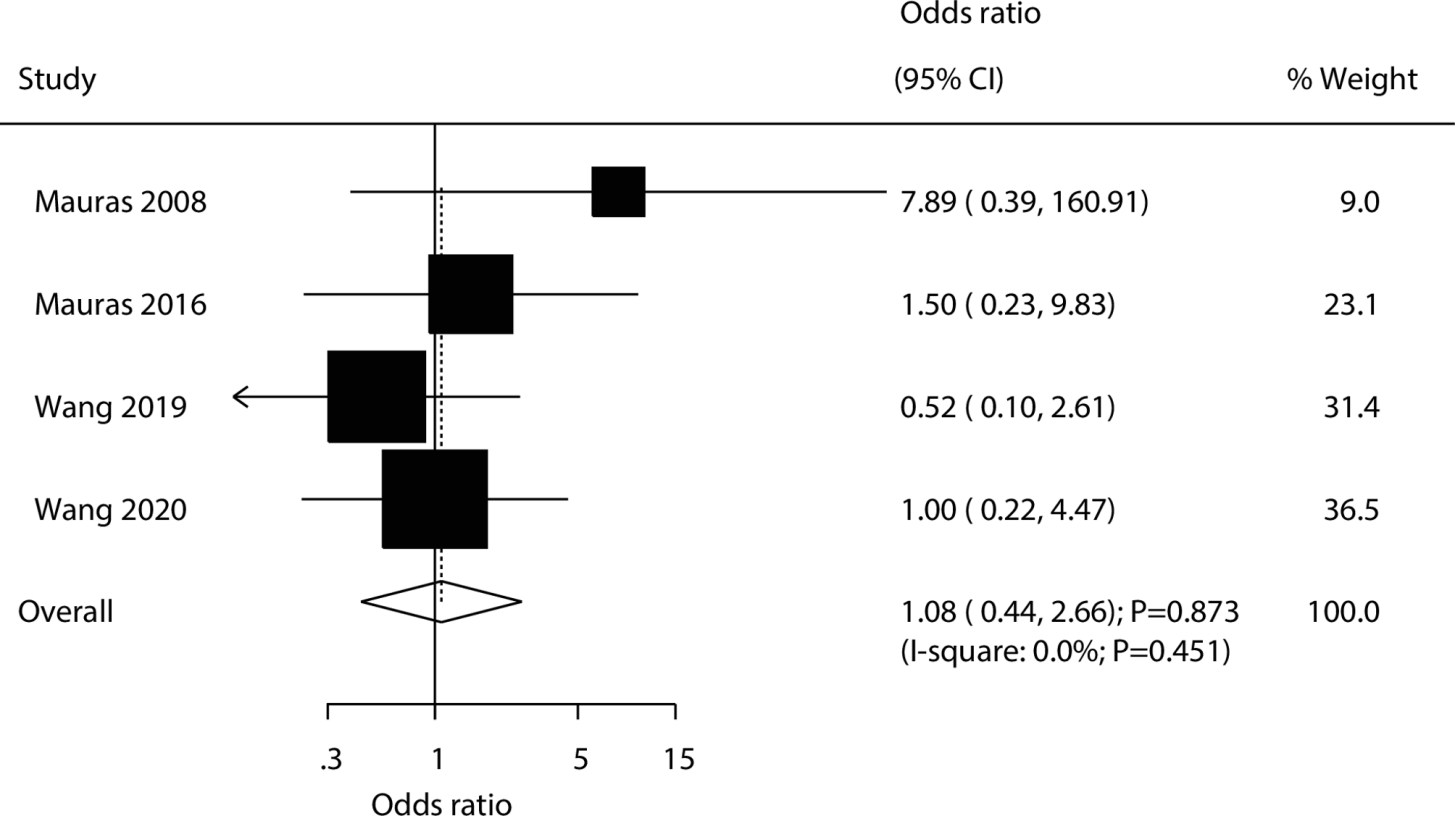
Effect of the combination of AIs and rhGH versus rhGH alone on the risk of adverse events

A total of four trials reported the effect of the combination of AIs and rhGH versus rhGH alone on the risk of adverse events. There were no significant differences between the addition of AIs to rhGH and rhGH alone for the risk of adverse events (OR: 1.08; 95% CI: 0.44–2.66; *P* = 0.873; Fig. 6), and no evidence of heterogeneity was observed (*I*^*2*^ = 0.0%; *P* = 0.451).

## Discussion

This study was the first meta-analysis to assess the effects of the combination of AIs and rhGH versus rhGH alone for male children and adolescents with short stature. A total of eight RCTs that included 433 participants were selected. We noted that the combination of AIs and rhGH versus rhGH alone showed additional benefits on growth velocity, predicted adult height, and bone age, while the differences between the groups for IGF-I, serum estradiol, serum testosterone, and adverse events were not associated with statistically significant. Moreover, the effects of the combination of AIs and rhGH versus rhGH alone on the predicted adult height may have been affected by publication year and type of intervention.

A Cochrane review that was performed in 2015 identified four RCTs and found that the use of AIs could improve short-term growth outcomes, while the final adult heights showed no improvement [27]. Liu et al. performed a meta-analysis of 14 studies and found that the use of AIs increased final height and predicted adult height significantly, while the changes in bone age and the BMD between the groups showed no statistically significant associations. Moreover, the use of AIs may increase the testosterone level, reduce the estradiol, and increase the risk of adverse events [28]. However, these studies used both AI monotherapy and AI combined therapies, and the net effects estimated for the addition of AIs to rhGH and rhGH alone were not addressed. Therefore, we performed this systematic review and meta-analysis of available RCTs to compare the effects of the combination of AIs and rhGH with rhGH alone in male children and adolescents with short stature.

Our study found the addition of AIs to rhGH may yields additional benefit on growth velocity, predicted adult height, and bone age, and nearly all of included trials reported similar trends or conclusions. The use of AIs effectively blocks the conversion of androgens to estrogens, exhibiting significant selectivity and potency, particularly for postmenopausal women with breast cancer; moreover, comparable pharmacokinetic profiles have been observed in young males [29, 30]. The use of AIs can inhibit aromatase activity in peripheral tissues, thereby preventing the conversion of testosterone to estrogen, which effectively slows skeletal maturation in adolescents [31, 32]. The subgroup analyses showed that the predicted adult height between groups may have been affected by the publication year and the type of intervention. This may be explained by economics and the pharmacokinetics effects of the agents at various doses.

There were no significant differences between the groups in the changes of IGF-I, serum estradiol, serum testosterone, and adverse events. Several reasons may explain these findings: (1) the use of AIs yielded additional growth increase with no IGF-I level increase because the androgens contribute a direct effect on epiphyseal growth plate, mediated likely by the androgen receptor; [33, 34] (2) the effect of the addition of AIs to rhGH on estradiol was reduced and that on testosterone was increased in the short-term, while the effect of the combination of AIs and rhGH was restricted after the long-term use of AIs; [20] (3) when estrogen production decreases, it may trigger feedback regulation of the hypothalamic-pituitary-gonadal axis, but this regulation does not necessarily lead to a significant increase in testosterone levels, particularly during childhood and adolescence when the endocrine system is still developing, and (4) the risks of adverse events were comparable between the groups and not consistent with prior meta-analyses. Our study reported that serious adverse events were lower than expected because the statistical power was inadequate to detect potential differences [28]. 

Several shortcomings of our study should be acknowledged. First, all of the included studies were designed as RCTs; however, the trials had low to moderate quality. Second, several other metabolic parameters were reported in few trials, and the pooled conclusions were variable. Third, the heterogeneity across included studies were not fully explained by using a sensitivity and subgroup analyses, which could explained by the dose or type of interventions, and treatment durations. Fourth, since studies have shown that an increase in predicted height may not translate into an equivalent increase in adult height, in the planning stage, the effect of combined AIs and rhGH versus those of rhGH alone on adult heights should be assessed, whereas no study reported such outcome. Finally, there were inherent limitations of the meta-analyses of the published articles, including the inevitable publication bias and restricted detailed analyses.

## Conclusions

This study found that the addition of AIs to rhGH compared with rhGH alone may provide additional benefits for male children and adolescents with short stature, including growth velocity, predicted adult height, and bone age. Further large-scale RCTs should be performed showing the treatment effects of the combination of AIs and rhGH versus rhGH alone at various follow-up times and duration.

## Electronic supplementary material

Below is the link to the electronic supplementary material.

Supplementary Material 1

## Data Availability

The original contributions presented in the study are included in the article/Supplementary Material. Further inquiries can be directed to the corresponding author.
