# Supplementary material for: Aromatase inhibitors for short stature in male children and adolescents treated with growth hormone: a meta-analysis of randomized controlled trials
Source: BMC Pediatr. 2024 Dec 18;24:813. doi: 10.1186/s12887-024-05301-0 (PMC11656550; doi:10.1186/s12887-024-05301-0)
Supplement: Supplementary file 1 — Supplementary Material 1 [file 12887_2024_5301_MOESM1_ESM.docx]

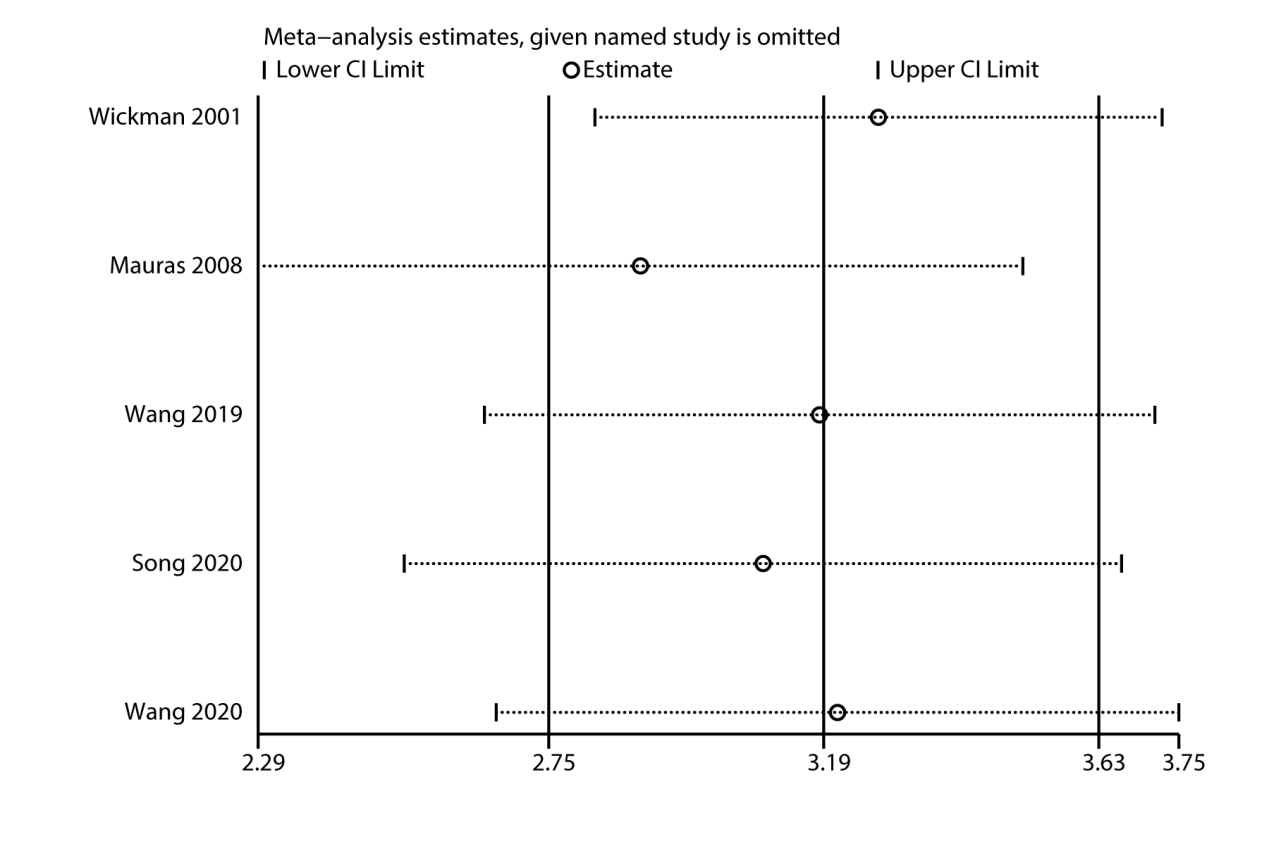


Figure S1. Sensitivity analysis for the effect of combined AIs and rhGH versus rhGH alone on the change of growth velocity.


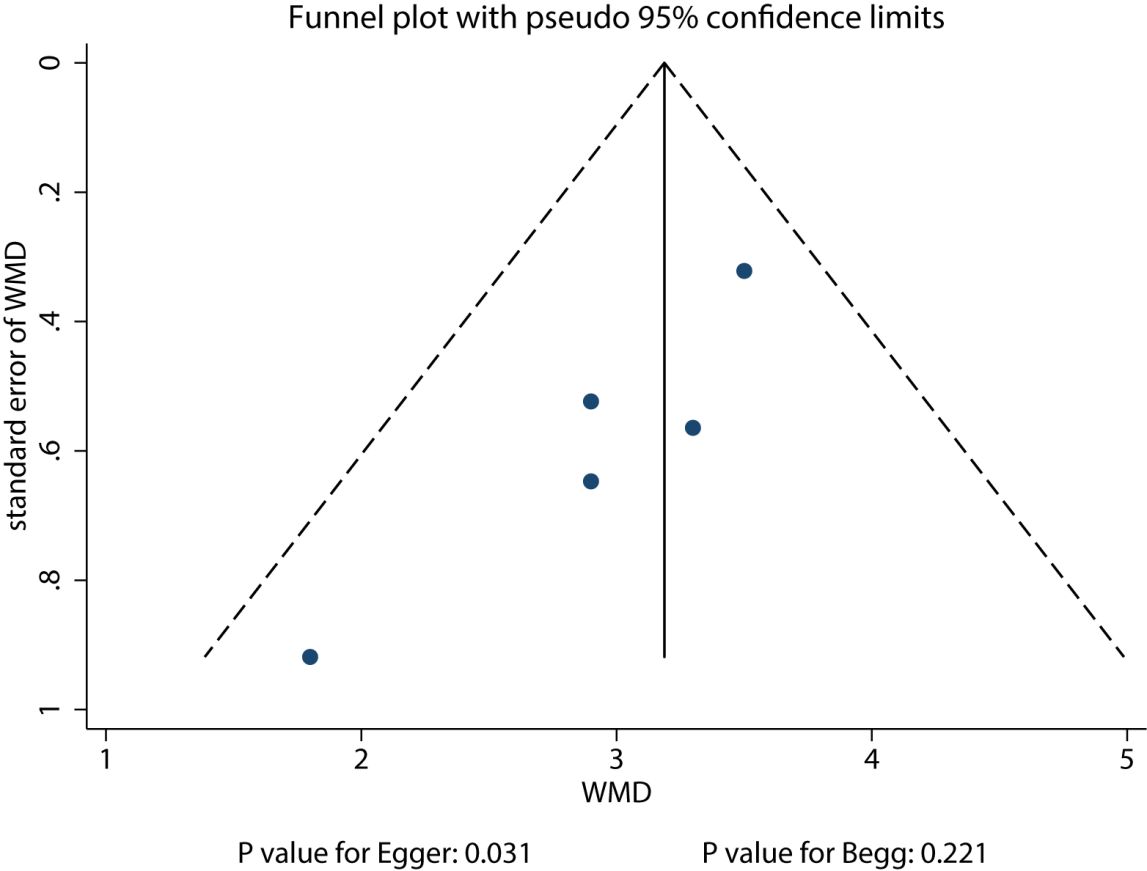


Figure S2. Funnel plot for growth velocity


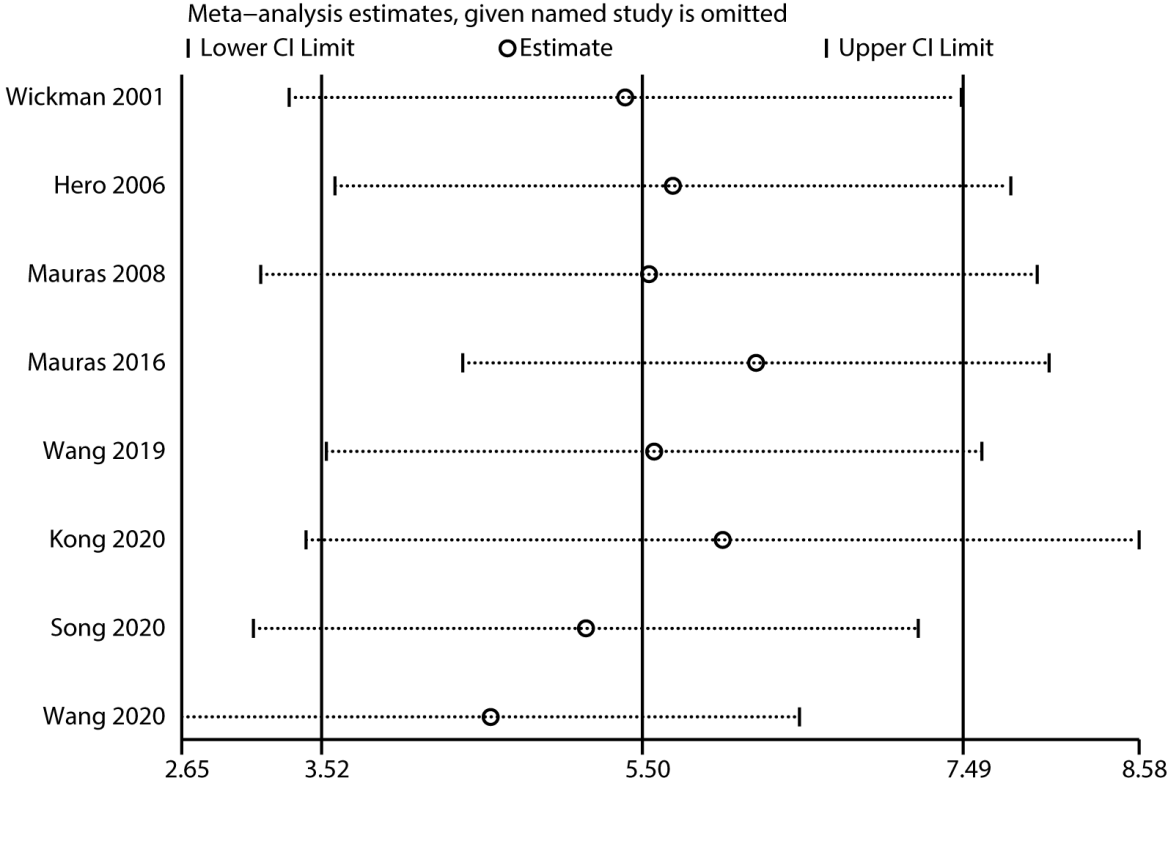


Figure S3. Sensitivity analysis for the effect of combined AIs and rhGH versus rhGH alone on the change of predicted adult height


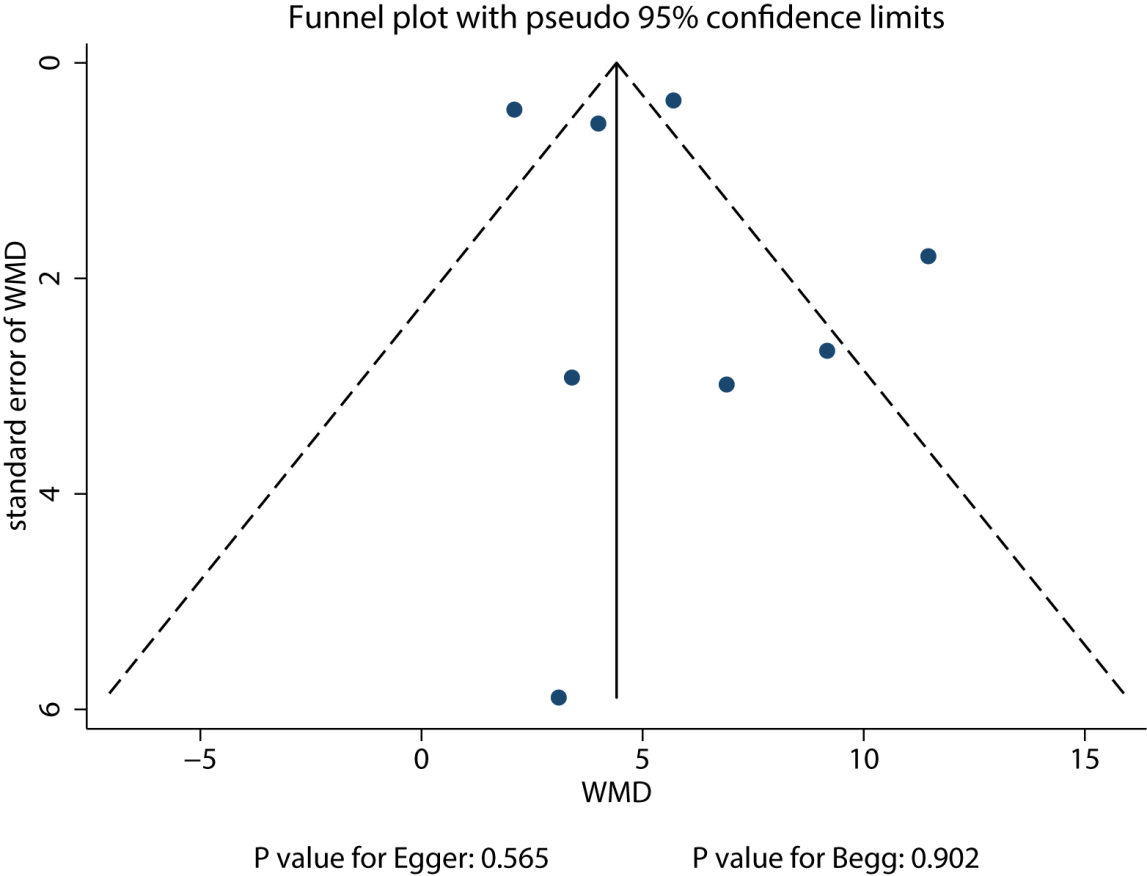


Figure S4. Funnel plot for predicted adult height


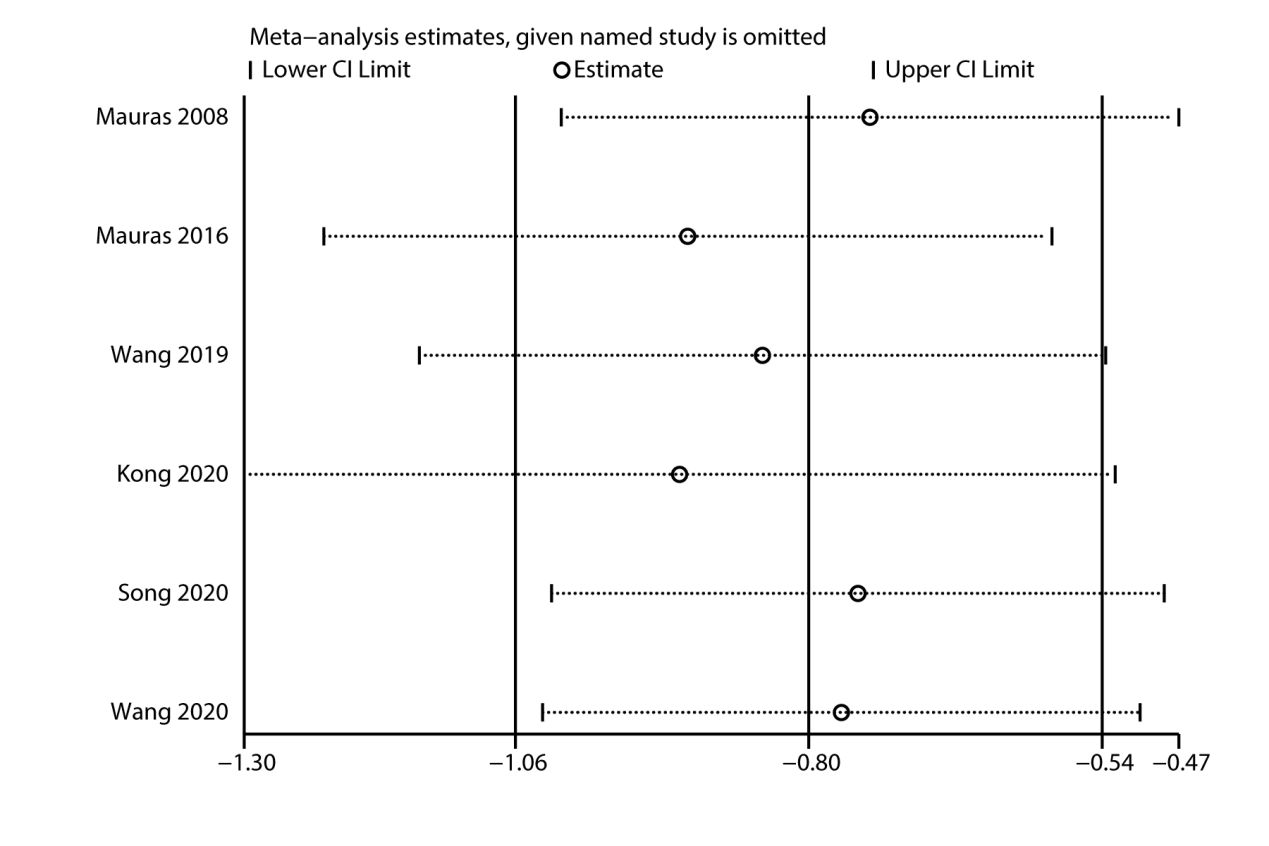


Figure S5. Sensitivity analysis for the effect of combined AIs and rhGH versus rhGH alone on the change of bone age


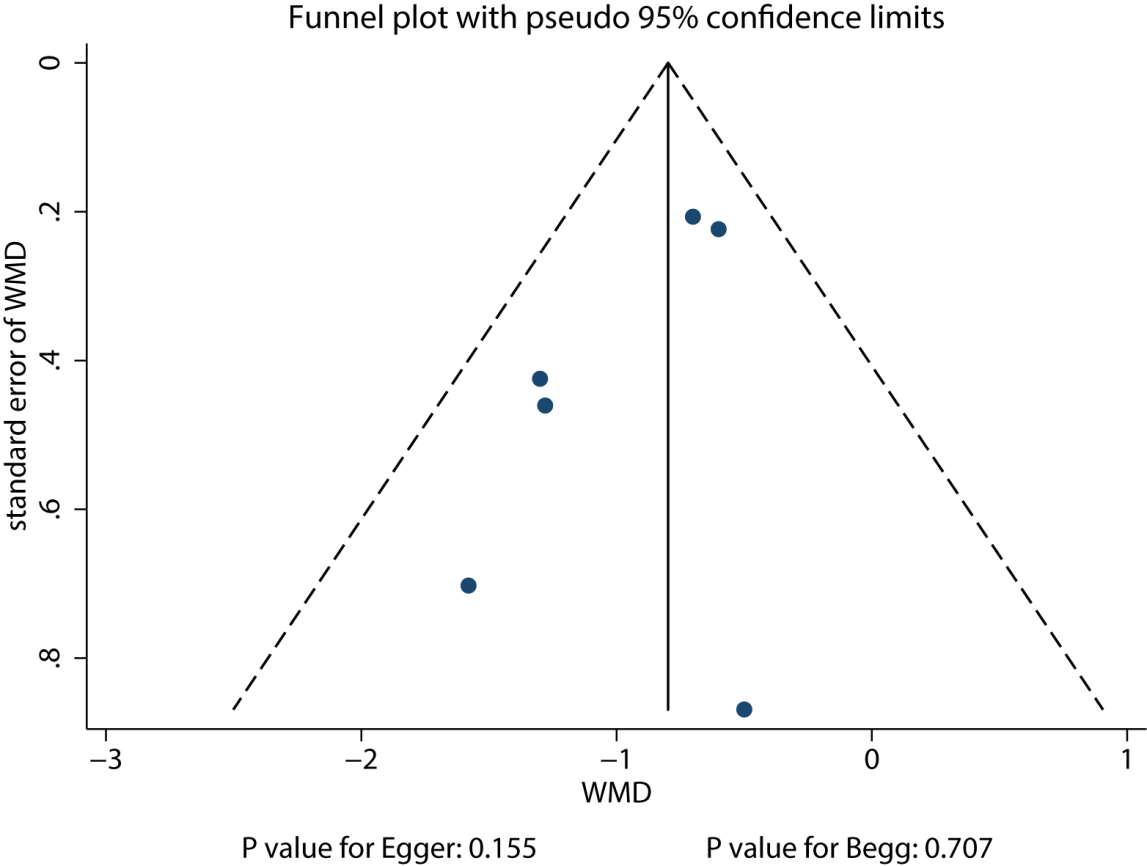


Figure S6. Funnel plot for bone age
